# Vegetarianism

**Published:** 1890-05

**Authors:** 


### VEGETARIANISM.

Atheroma is a peculiar degeneration of the arteries, which weakens them and renders them liable to rupture. In the most common form of apoplexy there is hemorrhage, the consequence of a breaking away of the walls of some of the vessels in the brain; and in the majority of cases the accident is due to atheroma. The causes of this degeneration are many, and include those influences which impair the general health and lower the tone of the system; and especially active in intemperance. There seems reason for believing that a purely vegetable diet conduces to atheroma. One disciple of vegetarianism, a physician, finds at the age of 40 that his arteries are becoming atheromatous to a marked degree, and he blames his diet for it. It appears that similar observations had been made, and the frequency of atheroma among the Hindoos is attributed to their vegetable diet. Considering the subject intelligently and impartially, but one conclusion can be reached; namely, that a mixed diet is the best for man.

